# Burnout in Italian Primary Teachers: The Predictive Effects of Trait Emotional Intelligence, Trait Anxiety, and Job Instability

**DOI:** 10.5964/ejop.2685

**Published:** 2022-05-31

**Authors:** Giacomo Mancini, Consuelo Mameli, Roberta Biolcati

**Affiliations:** 1 Department of Education Sciences “G.M. Bertin”, University of Bologna, Bologna, Italy; Maria Grzegorzewska University, Warsaw, Poland

**Keywords:** burnout syndrome, teachers’ wellbeing, trait emotional intelligence, anxiety trait, job instability

## Abstract

Burnout syndrome has recently been recognized as a public health problem, widely observed in educational settings. In this study, we aimed to examine the role played by contextual variables, including job (in)stability and teachers’ personal characteristics, in predicting factors associated with teacher burnout, using a convenience sample of 137 Italian primary school teachers (94.2% female, Age: M = 47.17, SD = 8.88). The findings from the hierarchical regression analyses showed that both trait emotional intelligence (EI) and trait anxiety predicted emotional exhaustion and lack of personal accomplishment in relation to work, with EI having a negative association and anxiety having a positive association with both. As for contextual variables, job instability positively predicted low personal accomplishment, whereas teachers’ working experience predicted emotional exhaustion. We discuss these results in light of the current working environment experienced by Italian teachers, which includes a high percentage of fixed-term workers. Moreover, we examine the implications for research and interventions related to trait EI as a protective factor that might prevent the onset of chronic professional burnout among teachers and increase teachers’ effectiveness and, therefore, pupils’ well-being, resulting in positive educational outcomes.

In the current education environment, teachers are required not only to effectively disseminate content and knowledge but also to demonstrate mastery of extensive emotional skills (Hughes, Luo, Kwok, & Loyd, 2008); promote positive relations with pupils (Mameli & Molinari, 2017), including those with special educational needs; and manage relationships with families, school colleagues, and the school principal. Given these conditions, it is not surprising that several national and international studies have documented a worrying increase in the occurrence of burnout syndrome among teachers (Aloe, Shisler, Norris, Nickerson, & Rinker, 2014; Murdaca, Oliva, & Nuzzaci, 2014).

## Theoretical Background

Traditionally, burnout has been described as a complex phenomenon involving three key components (Maslach, Jackson, Leiter, Schaufeli, & Schwab, 1986): Emotional exhaustion refers to the feeling of being physically and emotionally overwhelmed; low personal accomplishment concerns the tendency to negatively evaluate the value of one's work, a feeling of inadequacy in one’s own professional abilities, and generalized poor professional self-esteem; and depersonalization or cynicism indicates disengagement from and a distant attitude towards students. In general, burnout arises as a psychological response to the chronic work stress (Halbesleben & Demerouti, 2005; Schaufeli, Bakker, & Van Rhenen, 2009) associated with helping professions. Nevertheless, in recent years, researchers have extended the association of this phenomenon to educational and teaching professions as well, based on the understanding that they are also “emotional” jobs (Bhave & Glomb, 2016) that constantly require the adjustment of one’s own emotions to meet pupils’/students’ learning needs.

A number of studies have consistently shown that burnout is related to a broad range of negative outcomes, including depression, social withdrawal, and psychosomatic health problems (Hakanen & Schaufeli, 2012). It thus becomes critical to investigate which factors, both personal and contextual, could act to increase risk or protect against burnout. One line of such research is found in the theoretical framework of the job demands–resources (JD-R) model of burnout (see Demerouti, Bakker, De Jonge, Janssen, & Schaufeli, 2001; Hakanen, Schaufeli, & Ahola, 2008; Schaufeli & Salanova, 2014). In its original conceptualization, the JD-R model identified two broad categories of job-related characteristics as major antecedents of employees’ strain: job demands (i.e., workplace attributes related to physiological and psychological costs) and available resources (workplace features that function to assist employees in reaching work goals, diminishing job demands, and sustaining individual growth). More recently (e.g., Schaufeli & Taris, 2014), this model was expanded to include individual characteristics and dispositions among both personal demands (e.g., overcommitment) and resources (e.g., resiliency, self-efficacy, optimism). While a wide range of research addresses the effects of the balance between positive (resources) and negative (demands) job characteristics on employee health and well-being, only a few studies have investigated the role affective personal dispositions play in predicting different perceptions of job characteristics (Lavigne, 2014; Mazzetti, Schaufeli, Guglielmi, & Depolo, 2016).

Starting from these considerations and based on the latest version of the JD-R model of burnout, we aim to deepen understanding of the role of both individual characteristics and job-related stressors in burnout among Italian primary school teachers. We focus in particular on three dimensions: emotional intelligence (EI; which we consider an individual protective factor or resource), anxiety (which we consider an individual risk factor or demand), and job (in)stability (which can act as either a protective or a risk factor, a job demand or resource, depending on the type of teacher contract). In the following sections, we consider each of these variables in greater detail.

### Emotional Intelligence

Emotional jobs have been conceptualized as involving three key components (Tuxford & Bradley, 2015): exposure to emotionally demanding situations, emotional effort, and work focused on the emotional well-being of others. These kinds of demands require individuals to have several personal and emotional abilities, such as managing one’s own and other’s feelings and regulating emotions in interpersonal relationships, which are at the core of the EI construct. EI has recently become a crucial element in educational literature, as research has shown that it works as a protective factor supporting teachers’ mental well-being as well as teaching–learning processes (Molero, Ortega, Jiménez, & Valero, 2019).

Based on existing theoretical frameworks, EI might be defined as a wide array of affect-related individual differences that convey the adaptability characteristic of intelligence and the subjective experience of emotion. As highlighted by Hughes and Evans (2018), “EI-related characteristics can be considered constituents of existing models of cognitive ability (ability EI), personality (trait EI), and emotion regulation (EI competencies)” (p. 1). At school, teachers’ EI manifests itself when the teacher is effective in dealing with their own and pupil’s feelings and emotions, that is, they are able to understand and discriminate among different emotions and to generate and reflectively regulate affects in order to promote emotional awareness growth (Mortiboys, 2013). From this perspective, teachers’ EI involves both abilities (such as perceiving, recognizing, and managing emotions) and dispositions (such as self-awareness and self-motivation) that have a central role in handling interpersonal relationships, leading the class group, and dealing with parents and colleagues.

In the existing literature, EI has been operationalized according to two main theoretical frameworks, namely, the ability and trait models (see Petrides, 2010). The ability model (Mayer & Salovey, 1997) conceives of EI as the mental ability to perceive, use, understand, and manage one’s own and others’ emotions. The trait model (Petrides et al., 2016), consistent with theoretical models that describe EI as a lower order personality construct, conceptualizes EI as a constellation of emotional perceptions and a collection of affect-related personality traits. Trait EI (or emotional self-efficacy) essentially concerns individuals’ perceptions of their inner world.

Previous studies (for a review, see Mérida-López & Extremera, 2017) pointed out that trait EI is a relevant protective factor with respect to teachers’ burnout given its role in mitigating the impact of occupational stressors, preventing negative moods, facilitation the experience of positive emotional states, and enhancing stress resilience and well-being among teaching professionals. For instance, research focused on teacher efficacy in relation to the construct of EI (Vesely, Saklofske, & Leschied, 2013) showed how higher levels of EI can 1) mediate stress escalation and improve teachers’ management; 2) help facilitate effective teaching and well-being; and 3) contribute to a large portion of the positive factors linked to building resilience. The protective effect of EI has been recently confirmed in highly stressful scenarios such as those connected with the current Covid-19 pandemic, a disruptive event with devastating consequences for both workplaces and the global community (Hu, He, & Zhou, 2020). For instance, high levels of trait EI have been shown to predict a lower intensity of negative emotions (Moroń & Biolik-Moroń, 2021).

### Trait Anxiety

Acting as a counterpart of EI, anxiety—a psychological and physiologic condition characterized by cognitive, somatic, emotional, and behavioral components (Ahmed & Westra, 2009)—has been described as a risk factor for burnout (Ding, Qu, Yu, & Wang, 2014; Koutsimani, Anthony, & Georganta, 2019; Sun, Fu, Chang, & Wang, 2012). In fact, it may hinder the handling of an individual’s own affective reactions, as well as affective reactions in response to others, by worsening the quality of school life for teachers, pupils, and their parents.

Anxiety is commonly described with reference to two related constructs (Turnipseed, 1998): state and trait. State anxiety corresponds to the individual’s reaction toward a situation previously evaluated as threatening, while trait anxiety refers to an individual’s proneness to anxiety, which represents a stable personality dimension. On the one hand, work-related stressors can favor anxiety symptoms or states (DiGiacomo & Adamson, 2001). On the other, those individuals who are stably more introverted and more vulnerable to worries and overthinking are more likely to experience work-related frustration and exhaustion (Maslach, Schaufeli, & Leiter, 2001), emotional exhaustion, and cynicism (Ding et al., 2014), and high burnout levels as well (Vasilopoulos, 2012).

### Job Instability

Apart from the individual characteristics involved in the management of emotions and therefore of burnout, teachers in schools of any level are exposed to a number of potential contextual stressors (Grayson & Alvarez, 2008; Guglielmi & Tatrow, 1998; Klassen & Chiu, 2010; Skaalvik & Skaalvik, 2017; Yu, Wang, Zhai, Dai, & Yang, 2015), such as low administrative and colleague support, time pressure, lack of resources, and large class sizes. In addition to these known stressors, in recent years, another contextual factor has attracted scholars’ attention: occupational instability.

In Italy, like in other European countries, the austerity policies implemented following the economic crisis that started in 2008 have had an impact on the school system and its primary workers, teachers. All public administrations, schools included, have been subject to curbs on staff expenditure (Pirani & Salvini, 2015), with a significant decrease in recruitment and the stipulation of temporary contracts. According to a report published by ISTAT (Statistics National Institute) in 2017, the rate of fixed-term jobs for teachers in public schools was estimated at 14% for the academic year 2014/2015. Furthermore, in the last few years, news reports have recorded an alarming level of discontent among teachers, signaled by the many protests in public squares all over Italy, by teachers in precarious positions. Consequently, in July 2019, the EU Commission opened an infringement procedure against Italy for its excessive use of fixed-term contracts in public administrations, including schools.

The few existing studies on this matter (e.g., Aybas, Elmas, & Dündar, 2015; De Cuyper, & De Witte, 2008; Virtanen et al., 2005) suggest that fixed-term employment affects employees’ job attitudes and well-being, as job insecurity is positively associated to burnout syndrome and is a mental health issue. For example, a study by Bosman, Rothmann, and Buitendach (2005) on a sample of employees in a government organization, showed job insecurity was positively related to burnout. Despite this evidence, while contract-related stressors have been studied in particular in mental health professionals (Dreison et al., 2018; López‐López et al., 2019) and more broadly, in employees (Hünefeld & Köper, 2016), the literature in the educational field is still limited and, to our knowledge, no studies have explored the connection between job instability and burnout in the in-service primary teacher population in contexts with high levels of temporary employment.

The issue of insecure employment is not independent of the workers’ experience levels, given that less experienced and younger people are most likely to be in positions that are precarious. In Italy, 78% of teachers working in public schools are between 45 and 64 years old, meaning the “teaching workforce in Italy is the oldest among OECD countries” (OECD, 2019, p. 4). Research has reported inconsistent results on the impact of teachers’ experience on burnout levels. Some studies have indicated more experienced teachers are more at risk of burnout (Kokkinos, 2007), while others found novice teachers to be more vulnerable to emotional exhaustion (Boles, Dean, Ricks, Short, & Wang, 2000; Gavish & Friedman, 2010), and others still found no difference (Mameli & Molinari, 2017).

## Method

The aim of the current study was to analyze the role played by teachers’ trait EI and trait anxiety in predicting elements of teacher burnout, after controlling for job (in)stability and teaching experience. In line with the literature, we hypothesized that job instability would positively predict the burnout variables considered (Aybas et al., 2015). Since previous studies (Boles et al., 2000; Kokkinos, 2007) showed contradictory results on the association between teaching experience and burnout, no prediction was made about this point. Finally, and in line with previous investigations (Mérida-López & Extremera, 2017), we predicted that teachers reporting high EI would experience low levels of burnout, while teachers reporting high anxiety would experience high levels of burnout.

### Participants and Procedures

A survey was distributed to a convenient sample consisting of 137 Italian teachers, mostly females (94.2%), from six primary schools located in four medium-sized towns in North Eastern Italy. Their ages ranged between 28 and 66 (*M* = 47.17, *SD* = 8.88), while their teaching experience varied from 1 to 41 years (*M* = 20.15, *SD* = 10.51). As for their professional careers, teachers reported having worked on a fixed-term contract for 5.52 years on average (range: 0–27, *SD* = 4.67) and on an open-ended contract on average for 15.24 years (range: 0–37, *SD* = 11.05). At the time of survey administration, 36.5% (*n* = 50) of teachers declared they had a fixed-term contract.

Before teachers filled out the questionnaire, the study was presented to the school principals and teacher representatives for formal approval. The survey was available in an online and a print version (for teachers unfamiliar with computerized procedures). The items of the self-report measures were presented in the same order in both forms. In line with ethical norms set by the Italian National Psychological Association, both these forms were preceded by an introduction illustrating the general goal of the study and emphasizing data confidentiality and anonymity.

Thirty-two teachers (23.4 %) filled the in paper-version of the questionnaire, while the others completed the online form. We kept the survey open for 3 weeks, allowing teachers enough time to access the survey based on their other commitments.

### Measures

#### Demographics

Participants were asked to indicate the following on a sociodemographic data form: gender, age, type of employment contract, as well as their years of teaching experience. In order to investigate these last two variables, the following single-item were used: “*Is your employment contract open-ended or fixed-term?*” and “*How many years have you been working as a teacher?.*”

#### Teacher Burnout

This issue was assessed using the Maslach Burnout Inventory (MBI; Maslach & Jackson, 1981) in its validated Italian form (Sirigatti & Stefanile, 1992), with specific reference to teaching professionals. The MBI consists of 22 items grouped into three scales: emotional exhaustion (nine items, e.g., “I feel emotionally drained by my work”), personal accomplishment (eight items, e.g., “I can easily create a relaxed atmosphere with my students”) and depersonalization (five items, e.g., “I feel students blame me for some of their problems”). Teachers were asked to specify how often they experienced these feelings on a 7-point Likert-scale, ranging from 0 (Never) to 6 (Daily). For this study, Cronbach’s alphas were 0.75 for both emotional exhaustion and personal accomplishment, and only 0.22 for depersonalization. Due to the very low internal consistency, and in line with other studies with similar findings (Mameli & Molinari, 2017; Worley, Vassar, Wheeler, & Barnes, 2008), we decided to remove the depersonalization scale from further analyses.

#### Trait Emotional Intelligence

TEIQue self-report instrument (Petrides & Furnham, 2004), in its shortened 30-item form (Petrides, 2009), was used to measure global trait Emotional Intelligence (trait EI). The items in TEIQue-SF are either positive (e.g., “Expressing my emotions in words is not a problem for me”) or negative (e.g., “I often find it difficult to show my affection to those close to me”). Responses to the TEIQue-SF items are made on a 7-point Likert scale (range from 1 = strongly disagree and 7 = strongly agree). The total scale scoring is calculated by summing the score on each item in the scale (after reverse scoring for negative items) and is used to position respondents on the latent trait continuum: the higher the score, the higher the trait EI of the individual. The TEIQue-Short Form has proved to be a useful and reliable tool also in the Italian context (Di Fabio & Palazzeschi, 2011a, 2011b), allowing us to identify the four main dimensions of the trait EI in a quick and well-defined manner. In our sample, Cronbach’s alpha for this scale was 0.81.

#### Anxiety

The 20-items trait scale of the State-Trait Anxiety Inventory (STAI—Y; [51] Spielberger, Jacobs, Russell, & Crane, 1983), in its validated Italian version (Pedrabissi & Santinello, 1996), was used to evaluate teachers’ anxiety issues. Sample items for this scale are “I worry too much over something that doesn’t really matter” and “I am a level-headed person” (reversed). Teachers were asked to indicate the frequency with which they experience these feelings on a 4-point Likert-scale ranging from 1 (Almost Never) to 4 (Almost Always). Cronbach’s alpha for this scale was 0.92.

## Results

Before testing our hypotheses, we computed descriptive statistics and intercorrelations among the variables (see Table 1).

**Table 1:** Descriptive Statistics For and Intercorrelations Among the Considered Variables

Variable	*M*	*SD*	Range	1	2	3	4
1. Emotional Intelligence	5.19	0.56	1-7	1			
2. Anxiety trait	1.82	0.43	1-4	-.545**	1		
3. Emotional exhaustion	1.62	0.89	0-6	-.332**	.432**	1	
4. Low personal accomplishment	1.85	0.80	0-6	-.384**	.369**	.153	1

**p < .01.

Significant, negative associations were found between EI and anxiety, emotional exhaustion and low personal accomplishment. Significant positive correlations were also found between anxiety and emotional exhaustion and low personal accomplishment.

Two separate hierarchical linear regression analyses were then conducted to estimate the effects of teachers’ EI and anxiety on each of the two burnout dimensions, emotional exhaustion and low personal accomplishment. We checked for the effect of teaching experience and job stability by entering them as dichotomic variables^1^1In order to dichotomize the teaching experience variables, we chose the median value of the years of teaching, which corresponded to 20. We then assigned a value of 0 to teachers with 1 to 20 years of experience (50.0%) and a value of 1 to the other teachers. For job stability, we assigned a value of 0 to teachers with a fixed-term contract and a value of 1 to those with open-ended contracts. in Step 1 of the regressions. Teachers’ EI and anxiety were included in the second step of each analysis.

Before going on with the analysis, the data were examined for outliers and for conformity with normal distribution. Using boxplots, we identified two outliers on the low personal accomplishment scale. These cases have been withdrawn from the data, reducing the sample size from 137 to 135 participants. For all the variables included in our regression models, values for skewness (range: −0.119 to 0.773) and kurtosis (range: −0.513 to 0.044) were lower than |1|, which advised that the distribution of the variables was adequate for the statistical analyses. To identify potential collinearity issues between the predictors, we computed a set of variance inflation factors (VIF; one for each of the two regressions). As the values of VIF (range; 1.030–1.410) were lower than the conventional threshold (10), it was determined that no multicollinearity issues existed.

The results of the two hierarchical regression analyses are presented in Table 2.

**Table 2:** Hierarchical Regression Analyses Summary for Job (In)Stability, Teaching Experience, Emotional Intelligence and Anxiety Trait Predicting Emotional Exhaustion and Low Personal Accomplishment

	Dependent Variables			
	Set 1 Emotional Exhaustion		Set 2 Low Personal Accomplishment	
**Step/Predictors**	**Step 1**	**Step 2**	**Step 1**	**Step 2**
Job (in)stability	-.05	-.08	.42***	.39***
Teacher experience	.25**	.28***	.03	.05
Emotional intelligence		-.18*		-.23**
Anxiety trait		.36***		.21*
*F*	.41**	13.06***	13.77***	15.24***
*R^2^*	.05	.27	.16	.31
*∆R^2^*	**-**	.23***	**-**	.15***

*p < .05. **p < .01. ***p < .001.

In the first set of analyses, related to emotional exhaustion, teaching experience (β = 0.25, *p* < .01) inserted in Step 1, explained 5% of the variance (adjusted *R*^2^ = .05). With respect to the two variables added in Step 2, EI (β = −0.18, *p* < .05) and trait anxiety (β = 0.36, *p* < .001) were both relevant predictors, in a positive and negative direction, respectively, for emotional exhaustion. Overall, the variables accounted for 27% of the variance (adjusted *R*^2^ = .27).

For the second set of analyses related to low personal accomplishment, job stability (β = −0.42, *p* < .001), inserted in Step 1, accounted for 16% of the variance (adjusted *R*^2^ = .16). In Step 2, job stability (β = −0.39, *p* < .001) and EI (β = −0.23, *p* < .01) were found to be significant negative predictors of the dependent variable, while trait anxiety (β = 0.21, *p* < .05) positively predicted it. A total of 31% of the variance (adjusted *R*^2^ = .31) was accounted for by the predictor variables.

## Discussion

The goal of the present study was to examine the role played by teachers’ EI and trait anxiety in predicting elements of teacher burnout, after controlling for job (in)stability and teaching experience, in a convenience sample of primary school teachers from Italy.

The findings from our descriptive and correlational analysis revealed that burnout levels reported in our sample were neither particularly high nor alarming, in line with other studies on the Italian teacher population (e.g., Mameli & Molinari, 2017). The high score reported for trait EI is encouraging and might contribute to interpreting the low average values on elements of burnout. In fact, in view of the many demands placed on teachers (Alarcon, 2011) and the well-known association between occupational stress and low job satisfaction (Paulík, 2012), it is possible that trait EI contributes to supporting the development of competencies that lead to improved psychological health and job-related satisfaction. Trait EI might be an essential personality construct in educational contexts because it encompasses facets (such as emotion expression, empathy, and assertiveness) that are relevant to interpersonal experience and teaching activities. Therefore, high-trait EI individuals are well equipped to regulate their emotional reactions over time, manage stress, and be assertive (Petrides & Furnham, 2001).

In addition, the negative association we found between trait EI and anxiety, which was in line with other studies (e.g., Dewaele, Petrides, & Furnham, 2008; Summerfeldt, Kloosterman, Antony, & Parker, 2006; Weaving, Orgeta, Orrell, & Petrides, 2014), seems to corroborate what has been described in the literature, namely that trait EI, which represents a stable personality trait, has a positive effect on emotion regulation (e.g., Kotsou, Nelis, Grégoire, & Mikolajczak, 2011; Laborde, Brüll, Weber, & Anders, 2011) that is incompatible with an anxious personality.

The findings from both sets of hierarchical regression analyses confirm our hypotheses, although not completely. First, as expected, job instability positively predicted low personal accomplishment (set 2 of the regression), while no association was found between this variable and emotional exhaustion. The extended periods of job insecurity that affect teachers on fixed-term contracts, combined with other emerging stress factors such as lack of career advancement opportunities and the diminished social prestige of the teaching profession, can lead to negative consequences for the individual’s perception of self-fulfillment at work. According to Del Valle, López, and Bravo (2007), individuals with the lowest employment stability perceive less support from the organization and have a more negative view of the conditions in which they work. These results align with those of Wade, Cooley, and Savicki (1986), who consider occupational instability a predictor variable of burnout. Moreover, although teachers in our sample had rather high levels of seniority of service, more than a third of them declared having a fixed-term job (see participants section). Even if this rate should be interpreted with caution, as our participants cannot be considered to be representative of the nationwide teaching population, it raises concerns about the Italian employment situation. Such a scenario could also lead to an increase in the perception of injustice and inequity among teachers in precarious positions who have not benefited, unlike others before them, from government decrees that have, in the past, allowed them to have long-term employment in schools.

A second finding showed that teacher experience positively predicted emotional exhaustion. As noted in the introductory section, the relationship between teaching experience and burnout is controversial (Bayram, Gursakal, & Bilgel, 2010; Egyed, & Short, 2006), so we made no assumptions about this result. Our data seem to align with another European study (Kokkinos, 2007), revealing a significant main effect of teaching experience on emotional exhaustion, with more experienced teachers showing greater emotional exhaustion than did novices. This is a noteworthy result as it highlights that the fatigue that can arise in working for a long time may have serious repercussions on a worker’s mental health. This finding raises concerns, especially given that Italy boasts teachers with the oldest average age in Europe (Valle, Normandeau, & González, 2015).

Finally, and in line with the literature (Frenzel et al., 2016; Mérida-López & Extremera, 2017) and in support of our third hypothesis, we found that trait EI and trait anxiety predicted emotional exhaustion and low personal accomplishment, in negative and positive directions, respectively. Nevertheless, our results provide important information as the strength of these associations was different for the different dimensions considered. In particular, trait anxiety was shown to be a key factor especially relative to emotional exhaustion, while trait EI was a stronger negative predictor for low personal accomplishment. These results have important implications in terms of prevention of teacher burnout. In fact, they seem to suggest that in order to prevent emotional exhaustion, risk factors such as anxiety should be addressed by providing teachers with psychological support, such as through group-based methodologies. Conversely, in regard to personal accomplishment, our data pointed out a more significant role for trait EI as a protective factor. This suggests that trait EI plays a key role in personal fulfilment at work, even more than it affects exhaustion, chronic tiredness, and demotivation related to burnout. In line with research that underlines the protective effect of trait EI regarding occupational stress (e.g., Mikolajczak, Menil, & Luminet, 2007), we assume that trait EI, as a personal affective disposition concerning individual differences in emotion-related self-perceptions, such as emotion regulation, adaptability, stress management and optimism, may support teachers by mediating job instability, allowing teachers to feel sufficiently fulfilled even in the context of temporary employment.

In concluding the discussion of our results, we cannot overlook the fact that our findings were taken from a sample almost entirely made up of female teachers. Although this fact represents one of the limitations of this study, it also reflects the reality of the Italian context. In fact, in Italy, among the various public services and agencies, education has the highest percentage of female workers. As reported in the 2016–2017 national survey (MIUR, 2018), 96.4% of teachers in primary school are women. This gender distribution is typical of the teaching profession in Italy as well as in other European countries such as Lithuania (97.1%), Hungary (97%), and Slovenia (96.9%), while in other North Europeans Countries (e.g., Denmark) teaching is more gender-balanced. Notwithstanding these considerations, we are aware that gender distribution may have affected the results of this study, as previous studies have indicated—sometimes with mixed results—regarding gender differences relative to EI (Schutte et al., 1998) and burnout in teachers (Rumschlag, 2017).

## Conclusions

The findings presented in this study have some limitations, which should be taken into account when interpreting the results. The data rely on a small sample, which might not allow the results to be generalized. In particular, the sample was solely Italian, limited to northern Italy, and consisted only of primary school teachers. Future research, conducted with larger populations of middle and high school teachers, is necessary to confirm the results of this study.

Second, the data are based on the participants’ perceptions, as they were collected via self-report. Although the measures we used have been broadly validated and are widely used among scholars as they are considered very reliable tools, perceptions do not necessarily correspond to actual feelings and actual abilities. Teachers may have under- or overestimated their anxiety or trait EI. Future research should use different methodologies, including observation, to assess the level of trait EI expressed in actual teaching practices.

Third, our study was focused on the variables that we chose to consider. We are aware that other variables might help explain and clarify the associations we found. Future lines of research should focus on other job and environmental factors potentially involved in the onset of burnout. For instance, looking at the current Covid-19 pandemic scenario, which puts all the school staff in a highly stressful situation, conducting research investigating strategies to avoid teacher burnout would be valuable, such as research investigating the protective role of EI against burnout. In addition, because individuals appear to respond differently to their environments, the role of personality differences should also be fully explored. For example, individual characteristics like creative thinking, openness, or the ability to produce humor might significantly and differentially affect the experience of teacher burnout. The ability to meet job demands using the internal resources of problem solving and/or creativity mitigates burnout as the consequence of job requests (Derakhshanrad, Piven, & Zeynalzadeh, 2019). However, Probst, Stewart, Gruys, and Tierney (2007) suggested that job insecurity may have adverse effects on creativity, yet moderately beneficial effects on productivity. For these reasons, it is important to investigate more thoroughly the mutual association and influences between all these variables.

Finally, as burnout syndrome can decrease the quality of the education delivered by teachers, which negatively affects a school’s excellence and pupil wellbeing, future studies may use longitudinal rather than cross-sectional research designs to explore the impact of teacher stress on teacher–pupil interaction and classroom climate. Moreover, intervention programs with a pre–post-test research design could be used to evaluate the benefits of trait EI training for enhancing teachers’ psychological well-being.

Despite these limitations, the current research emphasizes the roles of some individual (trait EI and trait anxiety) and contextual (job instability) variables as protective or vulnerability factors related to burnout in Italian primary school teachers.

We should bear in mind that teacher burnout is a serious condition that causes a high rate of attrition in the educational profession and negatively affects the quality of children's school experience. As for practical recommendations, combined training to control risk factors (such as excessive anxious stress) and enhance protective factors, such as trait EI, might be a promising way to support primary school teachers and reduce burnout, while improving their resilience and decreasing their stress and job dissatisfaction at the same time. Along these lines, as the core factors that define teacher efficacy are part of the competencies comprising trait EI, and as emotions are enmeshed with all aspects of the teaching–learning process (Pyhältö, Pietarinen, & Salmela-Aro, 2011), training interventions specifically aimed at developing trait EI in teachers would not only have considerable benefits for all teaching staff, but they could also positively impact teacher practices in the classroom and teacher–pupil relations.
